# Hempseed increases gamma-tocopherol in egg yolks and the breaking strength of tibias in laying hens

**DOI:** 10.1371/journal.pone.0217509

**Published:** 2019-05-28

**Authors:** Miloš Skřivan, Michaela Englmaierová, Tomáš Vít, Eva Skřivanová

**Affiliations:** Department of Nutritional Physiology and Animal Product Quality, Institute of Animal Science, Prague-Uhrineves, Czech Republic; University of Illinois, UNITED STATES

## Abstract

The effect of hempseed in the diet of laying hens was evaluated at 0, 30, 60 and 90 g/kg concentrations. The aim of the study was to determine the effect of dietary treatment on the performance of hens, the physical characteristics of egg quality, the concentrations of α- and γ-tocopherol and the carotenoid and cholesterol contents of egg yolks, together with the breaking strength of tibial measurements. In light of the obtained results, our study aimed to address the optimal dietary level of hempseed in laying hen diets. Lohmann Brown hens (n = 240) were divided into 4 dietary treatment groups (6 cages per treatment) with 10 hens per cage. The experiment lasted for 12 weeks. The level of nutrients in all diets (wheat-corn) was well balanced. The dietary metabolisable energy was adjusted using rapeseed oil. The addition of 30 g/kg of hempseed to the diet significantly increased (P˂0.001) egg production and egg mass. Alpha-tocopherol increased significantly (P = 0.002) only in the case of the 60 g/kg hempseed level (101 mg/kg dry matter (DM) versus 83 mg/ kg DM in the control group). In contrast, the level of γ-tocopherol increased gradually from 11 mg/kg DM in the control to 29, 39 and 43 mg/kg DM at the 30, 60 and 90 g/kg levels of dietary hempseed, respectively. The concentrations of beta carotene, zeaxanthin and lutein in egg yolks were not influenced by the dietary treatment. Hempseed at 90 g/kg (P = 0.036) decreased egg shell thickness without affecting its strength. The addition of hempseed decreased (P˂0.001) the cholesterol concentration in the egg yolks in all experimental groups. The addition of 30, 60 and 90 g hempseed increased (P˂0.001) the breaking strength of the tibia to 354, 352 and 350 N, respectively, compared to 297 N in the control group. The highest level of hempseed in the diet positively influenced the Ca concentration in the tibia (P = 0.021). The concentration of P in the tibia was negatively affected in the 60 to 90 g/kg hempseed treatments (P˂0.001). Eggs are a significant source of α-tocopherol. Based on our results, there is a possibility for enrichment of egg yolks with γ-tocopherol, with all of its associated health benefits, by the addition of hempseed to the diet of laying hens. Another significant benefit of hempseed is its effect on the breaking strength of the tibia, which can help with crucial problems in the commercial breeding of laying hens.

## Introduction

Hemp (*Cannabis sativa* L.) has been known for its bioactive substance composition, including tocopherols. Tocopherols can lower the risk of cardiovascular diseases, cancers and aged-related macular degeneration, among other physiological effects [1]. In hempseed, the concentration of tocopherols, as well as the fat content and fatty acid profiles, may vary according to the cultivar, as demonstrated by Kriese et al. (2004) [2], who measured the fat content, fatty acid profiles and tocopherol concentrations of 51 hemp genotypes over 2 years. In the hempseed, the predominant tocopherol was identified as γ-tocopherol (21.68 mg/100 g), followed by α- (1.82 mg/100 g), δ- and β-tocopherol [2, 3]. According to Jiang et al. (2001) [4], γ-tocopherol is very easily absorbed and accumulates in human tissues. It is metabolised mostly by 2,7,8-trimethyl-2-(beta-carboxyethyl)-6-hydroxychroman (γ-CEHC) and is excreted mainly through urination. Both γ-tocopherol and γ-CEHC, but not α-tocopherol, have some anti-inflammatory properties related to cyclooxygenase activity inhibition [4]. Gamma-tocopherol is somewhat less potent in donating electrons than α-tocopherol and is thus a slightly less powerful antioxidant [5]. Therefore, α-tocopherol is generally considered to be more potent than γ-tocopherol as a chain-breaking antioxidant for inhibiting lipid peroxidation. However, the unsubstituted C-5 position of γ-tocopherol appears to make it better able to capture lipophilic electrophiles, such as reactive nitrogen oxide species (RNOS) [6]. In addition, animal studies generally showed positive effects of α-tocopherol supplementation on bones in various models of osteoporosis. However, in high doses, it may be harmful to bone [7]. α-Tocopherol supplementation tended to induce an osteogenesis-dominant bone mass increase in the vertebral secondary cancellous bone, in which active bone remodelling occurs. Therefore, α-tocopherol consumption may have beneficial effects on bone health [8]. Additionally, Borhanuddin et al. (2012) [9] showed that α-tocopherol may have a significant effect on bone formation during the normal remodelling phase of secondary bone healing. On the other hand, Hamidi et al. (2012) [10] found that vitamin E supplements in the form of α-tocopherol suppressed serum γ-tocopherol levels and had negative effects on bone formation. In contrast to α-tocopherol, dietary supplementation with γ-tocopherol leads to not only an increase in γ-tocopherol itself but also an increase in α-tocopherol in the blood [4]. Another bioactive compound related to bone metabolism is cannabidiol. Cannabidiol enhances fracture healing by targeting collagen crosslinking [11]. A positive effect of *Cannabis sativa* extracts on the morphology and growth of bone marrow mesenchymal stem cells in rats was reported by Sazmand et al. (2018) [12]. Regarding experiments on poultry, the effects of both hempseed and hemp oil on the performance of laying hens and the fatty acid profiles of their eggs have been studied thus far. The results of Goldberg et al. (2012) [13] show that hemp used in hen diets led to an increased omega-3 polyunsaturated fatty acid content and colour intensity of egg yolks and did not have adverse effects on the sensory profiles of the cooked eggs. As demonstrated by Neijat et al. (2014) [14], both hempseed and hemp oil (10% and 4.5% in the laying hen diet, respectively) are well tolerated, safe and effective. Hemp pomace supplementation in the diet of hens in a final concentration of up to 10% enriched egg yolks with polyunsaturated fatty acids, with no negative effects on hen performance [15].

On the basis of a lack of studies concerning the effect of hempseed on bone quality and inconsistent results related to tocopherol and bone formation, the experiment with graded doses of hempseed from 30 to 90 g/kg of wheat-soybean meal based diet for laying hens was conducted. The experiment will determine which of these doses is best in terms of the performance of the hens. Furthermore, the experiment will show how this effective dose of hempseed increases the concentration of γ-tocopherol in egg yolks and increases tibia strength. Thus, the aim of the study was to evaluate the effects of different hempseed concentrations in hen diets on the performance of hens, physical characteristics of eggs, concentrations of γ-tocopherol, α-tocopherol, carotenoids and cholesterol in egg yolk, and parameters of tibia quality.

## Material and methods

### Hens, husbandry and diets

Two hundred forty 52-week-old Lohmann Brown (Egg Production Company in Kosičky, Kosičky, Czech Republic) hens were randomly assigned to 4 treatments with 6 replicate cages and 10 hens per cage. The hens were housed in three-floor enriched cages in the same air-conditioned facility. Each cage was 7,560 cm^2^ in area. The cage equipment conformed to the European Council Directive 1999/74 EC (European Union 1999). The light cycle was 16 h of light and 8 h of darkness, and the light intensity was approximately 10 lux in the central storey. The control diet had no hempseed addition, and the experimental groups received diets supplemented with 30, 60 and 90 g of hempseed per kg of diet. The ingredients and nutrient composition of the diets are listed in Table 1. All diets were formulated to contain similar levels of metabolisable energy (11.6 MJ/kg) and crude protein (165 g/kg). A vitamin-mineral premix incorporated into the diet did not contain tocopherol. Feed and fresh water were supplied *ad libitum*. The experiment lasted 12 weeks. The Ethical Committee of the Institute of Animal Science approved the study protocol and the use of animals in the experiment.

**Table 1:** Composition of hen diets and hempseed.

Ingredient (g/kg)	Hempseed in diets (g/kg)				Hempseed
	0	30	60	90	
Wheat	440.0	440.0	440.0	440.0	
Maize	161.7	147.7	133.7	120.7	
Soybean meal	230.0	225.0	220.0	215.0	
Rapeseed oil	35.0	28.0	21.0	14.0	
Wheat bran	25.0	21.0	17.0	13.0	
Hempseed	0.0	30.0	60.0	90.0	
Monocalcium phosphate	8.0	8.0	8.0	7.0	
Sodium chloride	3.0	3.0	3.0	3.0	
Coarse graded limestone, 1–2 mm	91.3	91.3	91.3	91.3	
DL-Methionine	1.0	1.0	1.0	1.0	
Vitamin-mineral premix^a^	5.0	5.0	5.0	5.0	
**Nutrient content (g/kg)**					
Dry matter	893.4	892.9	893.7	893.9	907.4
AME_N_ by calculation, MJ/kg	11.6	11.5	11.7	12.0	16.9
Crude protein	169.1	165.8	168.7	169.8	262.4
Ether extract	43.3	41.6	44.8	48.3	326.5
Crude fibre	34.5	34.1	36.9	39.7	125.9
Calcium	36.7	36.4	35.6	35.2	3.8
Phosphorus	4.3	4.3	4.4	4.6	11.2

^a^Vitamin-mineral premix provided per kg of mixed diet: retinyl acetate, 3.0 mg; vitamin D_3_, 3000 IU; niacin, 25 mg; Ca pantothenate, 8 mg; thiamine, 2.0 mg; riboflavin, 5 mg; pyridoxine, 4 mg; folic acid, 0.5 mg; biotin, 0.075 mg; cobalamin, 0.01 mg; choline Cl, 250 mg; menadione, 2.0 mg; betaine, 100 mg; butylated hydroxytoluene, 7.5 mg; ethoxyquin, 5.6 mg; butylhydroxyanisole, 1 mg; DL-methionine, 0.7 g; Mn, 70 mg; Zn, 50 mg; Fe, 40 mg; Cu, 6 mg; I, 1 mg; Co, 0.3 mg; Se, 0.2 mg.

AME_N_ = apparent metabolisable energy

The health status of the hens and the number of eggs were monitored daily. Hen-day egg production and feed intake were calculated weekly on a per-cage basis. Egg weights were determined three times per week.

### Analyses

The feed dry matter content was determined by drying in an oven (Memmert ULM 500; Memmert, Schwabach, Germany) at 105°C to a constant weight. The feed crude protein content was measured using a Kjeltec Auto 1030 instrument (Tecator, Höganäs, Sweden). The ether content in the diet was determined by extraction with petroleum ether using a Tecator 1045 Soxtec Extraction Unit (Tecator). Ash was determined from dry homogenised diets heated to 550°C, and the ash was dissolved in 3 M hydrochloric acid. The feed total P was analysed using a vanadate-molybdate reagent (AOAC International 2005; method No. 965.17). The feed Ca and Mg concentrations were measured in the hydrochloric acid extract by atomic absorption spectrometry using a ContrAA 700 F instrument (Analytik Jena AG, Jena, Germany).

For the physical parameter determinations, eggs were collected three times during the experiment; once within each collection period, whole-day egg production was analysed. A total of 642 eggs were analysed. The albumen, yolk, and shell percentages were determined based on the individual weight of each egg and the weights of its components. The albumen height was measured using an IP54 digital micrometer (Swiss Precision Instruments, Inc., Garden Grove, USA). Haugh units (HU) were calculated according to the methods of Haugh (1937) [16]. The shell breaking strength was determined on the vertical axis using an Instron 3360 apparatus (Instron, Norwood, MA, USA). After removing the shell membranes, the shell thickness (i.e., the average of 3 values from the sharp and blunt ends and equator of the shell) was measured using a micrometer. The eggshell index (SI) was calculated as follows [17]: SI = (SW/S) × 100, S = 4.68 × EW2/3, where SW = shell weight, S = shell surface, and EW = egg weight. The yolk colour was determined using the DSM yolk colour fan (DSM Nutritional Products, Basel, Switzerland) and Minolta CR-300 colorimeter (Konica Minolta, Osaka, Japan). The a* and b* parameters correspond to the redness (–100 = green, 100 = red) and yellowness (–100 = blue, 100 = yellow), respectively.

For the determination of cholesterol in yolks (n = 16), lipids were saponified, and the unsaponified matter was extracted with diethyl ether in accordance with ISO 3596:2011. Silyl derivatives were prepared using TMCS and HMDS silylation reagents (Sigma-Aldrich, Prague, Czech Republic) and quantified on a gas chromatograph equipped with a SAC-5 capillary column (Supelco, Bellefonte, USA) that was operated isothermally at 285°C.

The concentrations of α-tocopherol, γ-tocopherol and β-carotene in the feed and lyophilised yolks (n = 16) were determined after saponification and diethyl ether extraction in accordance with the EN 12823–1 and EN 12823–2 European standards.

The contents of lutein and zeaxanthin in the feed and lyophilised yolks (n = 16) were measured by high-performance liquid chromatography (HPLC) according to a modified method by Froescheis et al. (2000) [18] with the use of an HPLC instrument (VP series; Shimadzu, Kyoto, Japan) equipped with a diode-array detector. A wavelength of 450 nm was selected for detection. A Kinetex C18 column (100 × 4.6 mm; 2.6 μm) (Phenomenex, Torrance, USA) was used. A gradient system was established with acetonitrile:water:ethyl acetate (88:10:2) as eluent A and acetonitrile:water:ethyl acetate (88:0:15) as eluent B. The calibration standards Lutein and Zeaxanthin were supplied by Sigma-Aldrich (Prague, Czech Republic). The analysis duration was 18 min.

At the end of the experiment, sixteen hens from each treatment were slaughtered using CO_2_-based equipment for the euthanasia of poultry (Anieut G.d., Hena s.r.o., Miličín, Czech Republic). The tibia bones were excised from the carcasses and cleaned of all tissue. The breaking strength of fresh and boiled (1 h) tibias was measured using an Instron 3342 apparatus (Instron) with a 50-kg load cell with a crosshead speed of 50 mm/min. Each tibia was supported on a 5.75-cm span. The broken, boiled tibias were later used for other measurements. The bones were dried at 105°C for 72 h, placed in a desiccator, and weighed to determine their dry weight. Then, the bones were homogenised and placed in a muffle furnace at 600°C for 24 h and cooled in a desiccator, and the ash weight was recorded. The P, Ca and Mg contents of the tibia ash were determined using the same method as described for the analysis of these elements in the hens’ diets.

### Statistical analyses

The data were analysed using analysis of variance (ANOVA) with the general linear model (GLM) procedure using SAS software (SAS v 9.3, 2003) [19]. A one-way analysis of variance was used. The main effect was the dose of hempseed in the diet. All differences were considered significant at P < 0.05. The results in the tables are presented as the mean ± standard error of the mean (SEM).

## Results

The diet compositions are shown in Table 1. The diet enriched with hempseed at a 30 g/kg concentration significantly (P ˂ 0.001) increased both the egg mass and egg production (Table 2).

**Table 2:** Performance characteristics of hens.

Hempseed (g/kg)	0	30	60	90	SEM	Probability
**Hen-day egg production (%)**	88.7^b^	93.6^a^	86.4^b^	89.3^a^^b^	0.59	˂0.001
**Egg weight (g)**	63.6^c^	64.6^a^^b^	65.0^a^	64.4^b^	0.07	˂0.001
**Egg mass (g/day/hen)**	56.4^b^	60.5^a^	56.1^b^	57.5^b^	0.39	˂0.001
**Feed intake (g/day/hen)**	127.4^a^^b^	132.3^a^	125.3^b^	124.3^b^	0.75	0.001
**Feed intake (g/egg)**	144.4	141.6	145.2	140.1	1.08	NS
**Feed conversion ratio (g/g)**	2.27	2.19	2.24	2.17	0.016	NS
**Mortality (%)**	0	0	0	0		

^a-c^Means in the same row with different superscripts differ significantly.

The physical characteristics of the egg quality are shown in Table 3. Hempseed (30 g/kg) enhanced egg yolk redness, while 90 g/kg hempseed had the opposite effect (P ˂ 0.001). Egg yolk yellowness increased (P ˂ 0.001) with the 30 and 60 g/kg hempseed supplementation. Despite the aforementioned changes, the egg yolk colour remained at rather low values. The highest concentration of hempseed decreased (P = 0.036) egg shell thickness with no adverse effects on its breaking strength.

**Table 3:** Physical characteristics of egg quality and cholesterol content in egg yolks.

Hempseed (g/kg)	0	30	60	90	SEM	Probability
**Haugh units**	80.7	80.1	81.8	81.6	0.38	NS
**Albumen percentage (%)**	63.5	63.3	63.6	63.5	0.12	NS
**Yolk percentage (%)**	26.2	26.4	26.2	26.3	0.11	NS
**Shell percentage (%)**	10.3	10.2	10.2	10.1	0.04	NS
**Shell thickness (μm)**	363^a^	357^a^^b^	357^a^^b^	350^b^	1.5	0.036
**Shell breaking strength (g/cm**^**2**^**)**	4195	4128	4126	4377	42.3	NS
**Shell index (g/100 cm**^**2**^**)**	8.81	8.73	8.79	8.69	0.03	NS
**Yolk colour**						
** DSM yolk colour fan**	3.76^b^	3.86^b^	4.06^a^	3.81^b^	0.035	0.022
Redness (a*)	2.09^b^	2.42^a^	2.27^a^^b^	1.60^c^	0.059	˂0.001
Yellowness (b*)	40.2^b^	41.7^a^	41.8^a^	39.1^b^	0.21	˂0.001
**Cholesterol (g/kg)**	12.2^a^	10.8^b^	10.7^b^	10.6^b^	0.17	˂0.001

^a-b^Means in the same row with different superscripts differ significantly.

As the hempseed concentration increased, the amount of α-tocopherol, β-carotene, lutein and zeaxanthin increased accordingly (Table 4). The amount of γ-tocopherol is almost twenty times higher than that of α-tocopherol in hempseed, which was correspondingly increased in the mixed feed. In the control diet, with no hempseed added, α-tocopherol was higher, which was also observed in the diet enriched with 30 g/kg hempseed. When 60 g/kg hempseed was added, both tocopherols were almost equally present, and in the case of the 90 g/kg hempseed diet, γ-tocopherol was present at a higher concentration when compared with α-tocopherol. Regarding the tocopherol concentrations in egg yolks, eggs of hens fed with the control feed contained mostly α-tocopherol at 83 mg/kg DM; the amount of γ-tocopherol was 11.3 mg/kg DM (Table 4). The ratio between the two tocopherols was 7.35. With the increase in hempseed in the diet, the ratio decreased from 3.2 to 2.6 to 2. Between groups, there was a significant difference in both the α- and γ-tocopherol concentrations (P = 0.002 a P < 0.001, respectively). There were no significant differences in the amounts of β-carotene, lutein and zeaxanthin between treatments. In contrast, all experimental diets significantly decreased (P < 0.001) the cholesterol concentration in egg yolks. There was no effect of the hempseed concentration on cholesterol concentration.

**Table 4:** Vitamins and carotenoid concentrations in diets, hempseed and egg yolks.

**Diets and hempseed**						
**Hempseed (g/kg)**	**0**	**30**	**60**	**90**	**Hempseed**	
α-Tocopherol (mg/kg)	14.1	18.8	17.6	20.3	11.7	
γ-Tocopherol (mg/kg)	8.8	15.0	17.6	25.2	211.7	
ß-Carotene (mg/kg)	0.028	0.054	0.058	0.063	0.299	
Lutein (mg/kg)	0.89	1.03	1.38	1.43	7.61	
Zeaxanthin (mg/kg)	0.52	0.65	0.88	0.92	3.99	
**Egg yolks**						
**Hempseed (g/kg)**	**0**	**30**	**60**	**90**	**SEM**	**Probability**
α-Tocopherol (mg/kg DM)	82.9^b^	94.0^a^^b^	101.0^a^	86.0^b^	2.01	0.002
γ-Tocopherol (mg/kg DM)	11.3^c^	29.0^b^	38.6^a^	43.3^a^	2.32	˂0.001
ß-Carotene (mg/kg DM)	˂0.01	˂0.01	˂0.01	˂0.01		NS
Lutein (mg/kg DM)	7.39	7.32	7.64	6.93	0.129	NS
Zeaxanthin (mg/kg DM)	3.57	3.48	3.57	3.32	0.063	NS

^a-c^Means in the same row with different superscripts differ significantly.

DM = dry matter.

The breaking strengths of raw tibias were increased significantly (P < 0.001) with all dietary concentrations of hempseed, with no difference between the experimental hempseed diet groups (Table 5). After cooking, 6 and 9% hempseed in the diet significantly increased the breaking strength compared to that of the control. Nine percent hempseed increased the Ca concentration (P = 0.021) compared to the control, while the amount of P decreased with the addition of 60 and 90 g/kg hempseed. The amount of Mg was not influenced by the experimental diets.

**Table 5:** Breaking strength and mineral content of tibia bones of hens.

Hempseed (g/kg)	0	30	60	90	SEM	Probability
**Breaking strength of fresh tibia (N)**	297^b^	354^a^	352^a^	350^a^	10.7	˂0.001
**Breaking strength of boiled tibia (N)**	268^b^	292^b^	343^a^	344^a^	12.6	˂0.001
**Tibia calcium content (g/kg DM)**	170.8^b^	179.2^a^^b^	185.4^a^^b^	189.6^a^	4.20	0.021
**Tibia phosphorus content (g/kg DM)**	158.7^a^	150.5^a^^b^	148.0^b^	146.1^b^	2.05	0.001
**Tibia magnesium content (g/kg DM)**	3.01	3.20	3.15	3.12	0.108	NS

^a-b^Means in the same row with different superscripts differ significantly.

DM = dry matter.

## Discussion

Egg yolks are a valuable source of α-tocopherol. However, the amount of γ-tocopherol in egg yolk is close to zero. Guinazi et al. (2009) [20] evaluated the amount of both tocopherols in fresh eggs from two restaurants and found concentrations of 8.26 and 11.27 mg/kg for α-tocopherol; γ-tocopherol concentrations were below the detection limit. In our experiment, the dietary addition of hempseed resulted in 94–101 mg/kg DM of α-tocopherol and 29–43.3 mg/kg DM of γ-tocopherol in egg yolks. The control group had 83 mg/kg DM of α-tocopherol and 11.3 mg/kg DM of γ-tocopherol in their egg yolks. There was no vitamin E in the vitamin-mineral premix of the experimental animals. Tocopherols in the diet originated solely from the dietary components and mostly from the rapeseed oil. According to our analysis, rapeseed oil contains 217 mg/kg of α-tocopherol and 286 mg/kg of γ-tocopherol. Therefore, the presence of γ-tocopherol and its concentration in egg yolks is related to the presence of plant oils in mixed feed, which in our case was mostly hemp oil and rapeseed oil. Hempseed diets contained more α-tocopherol than the control diet. As the hempseed concentration increased in the feed, the amount of both γ-tocopherol and α-tocopherol increased in the egg yolk. The increased consumption of γ-tocopherol in laying hens corresponded with the increased amount of both tocopherols in egg yolks. Similar results were observed by Jiang et al. (2001) [4] in blood. In our experiment, the concentration of γ-tocopherol in hempseed was 21.2 mg/100 g, which is in agreement with the average γ-tocopherol concentration of 21.7 mg/100 g, as determined by Kriese et al. (2004) [2] from 51 genotypes. The small size of hempseed (on average 4 x 2.7 mm in our experiment) is ideal for mixed feed preparation when combined with coarse parts of cereals. The presence of hempseed in the diet did not influence its intake in any sense. In our opinion, the limit is the higher amount of fibre in hempseed, which can be a limiting factor for its use. However, the addition of 30% hempseed or 9% hemp oil to the diet did not cause any adverse effects on performance or egg quality [14]. Halle and Schone (2013) [15] investigated the effects of hempseed cake (5, 10 and 15%) in a feed mixture and concluded that feed with up to 10% cake did not negatively influence hen performance. A similar conclusion was made by Neijat et al. (2014) [14] after evaluating 10, 20 and 30% dietary concentrations of hempseed. In our case, even 3% hempseed in mixed feed increased egg production and daily egg mass. Hempseed did not affect the carotenoid concentrations in egg yolks. The effect on egg yolk colour was significant; however, the commercial output of this finding is marginal. The numerical values are close to the findings of Goldberg et al. (2012) [13], who observed similar effects of hempseed on yellowness and redness using higher concentrations of hempseed. Contrary to Halle and Schone (2013) [15], we did not observe a higher egg white proportion with hempseed supplementation in our experiment. Regarding the effect of hempseed on cholesterol level, there are a number of bioactive substances that can be responsible for that particular output. In intensive poultry production systems, bone strength is undoubtedly one of the major issues we need to address. The high incidence of broken bones is observed among hens throughout the production period, and during depopulation, transport and shackling [21, 22]. The positive effect of hempseed on the bone strength of the tibia is in agreement with results found in recent human medical literature [11] and experiments on rats [12]. The former author suggested that bone structure morphology and mesenchymal bone cell growth were possibly affected by hempseed. Similar modes of action can also be considered in poultry. Additionally, collagen is present in mesenchymal stem bone cells. In terms of the effect on bone metabolism, cannabidiol (CBD) is considered to be the active substance in hemp. As shown by Gabet (2017) [11], cannabidiol enhances fracture healing by targeting collagen crosslinking. Cannabidiol is a phytocannabinoid derived from *Cannabis* species that is devoid of psychoactive compounds. The higher tibial strength in our experiment is in agreement with the higher Ca concentration in groups fed the diets enriched by hempseed.

## Conclusion

In conclusion, dietary hempseed supplementation increased γ-tocopherol in egg yolks and the breaking strength of tibia and decreased cholesterol content in egg yolks. In terms of performance and bone quality, the most suitable dose for laying hens is 30 g/kg of hemp seed in the diet.
